# Pyronaridine tetraphosphate is an efficacious antiviral and anti-inflammatory active against multiple highly pathogenic coronaviruses

**DOI:** 10.1128/mbio.01587-23

**Published:** 2023-08-15

**Authors:** Jeremy Ardanuy, Robert Johnson, Carly Dillen, Louis Taylor, Holly Hammond, Stuart Weston, Matthew Frieman

**Affiliations:** 1 Department of Microbiology and Immunology, Center for Pathogen Research, University of Maryland School of Medicine, Baltimore, Maryland, USA; Icahn School of Medicine at Mount Sinai, New York, New York, USA

**Keywords:** antiviral agents, coronavirus, preclinical drug studies

## Abstract

The coronavirus disease 2019 (COVID-19) pandemic, caused by severe acute respiratory syndrome coronavirus-2 (SARS-CoV-2), continues to be one of the largest dangers to human health around the world. The need for effective antiviral and anti-inflammatory treatments is still extremely high as newly emerging variants threaten the efficacy of currently used treatment options. Many compounds are effective at inhibiting SARS-CoV-2 infection *in vitro* but fail to recapitulate that efficacy *in vivo*. There is a major demand for antiviral drugs that are efficacious and broadly effective for the treatment of highly pathogenic coronaviruses including SARS-CoV-2, and its close relatives SARS-CoV-1 and Middle East respiratory syndrome coronavirus (MERS-CoV). One drug with the potential to join the small subset of broadly active antiviral compounds that is both efficacious *in vivo* and orally bioavailable is pyronaridine triphosphate, which has now been published to show both *in vitro* and *in vivo* efficacy in A549 cells and the K18-hACE2 mouse model, respectively, by functioning as a protease inhibitor of the SARS-CoV-2 papain-like protease (PLpro). In our studies, pyronaridine treatment resulted in significant improvements to lung inflammatory pathology, reducing pro-inflammatory cytokine and chemokine levels, and inhibiting weight loss seen in the mouse model associated with the severity of disease in three highly pathogenic coronavirus infection models, SARS-CoV-1, SARS-CoV-2, and MERS-CoV. Additionally, we found that pyronaridine treatment can safely and effectively be combined with currently used therapeutics molnupiravir and nirmatrelvir (main protease inhibitor component of Paxlovid) in a SARS-CoV-2 *in vivo* model, and there was evidence of a synergistic effect that further reduced viral titers, inflammatory lung pathology, and inflammatory cytokine and chemokine levels. These results indicate that pyronaridine represents an excellent potential therapeutic candidate for COVID-19 treatment individually, or in combination with other approved antivirals as well as a potential therapeutic option for the treatment of highly pathogenic coronaviruses such as SARS-CoV-1, MERS-CoV, and future coronaviruses yet to emerge.

**IMPORTANCE:**

Pyronaridine tetraphosphate is on the WHO Essential Medicine List for its importance as a widely available and safe treatment for malaria. We find that pyronaridine is a highly effective antiviral therapeutic across mouse models using multiple variants of severe acute respiratory syndrome coronavirus-2 (SARS-CoV-2), and the highly pathogenic viruses SARS-CoV-1 and Middle East respiratory syndrome coronavirus responsible for previous coronavirus outbreaks. Additionally, we find that pyronaridine additively combines with current COVID-19 treatments such as nirmatrelvir (protease inhibitor in Paxlovid) and molnupiravir to further inhibit SARS-CoV-2 infections. There are many antiviral compounds that demonstrate efficacy in cellular models, but few that show this level of impact in multiple mouse models and represent a promising therapeutic for the current coronavirus pandemic as well as future outbreaks as well.

## INTRODUCTION

Coronavirus disease 2019 (COVID-19), caused by severe acute respiratory syndrome coronavirus-2 (SARS-CoV-2), is a respiratory virus capable of causing lethal disease (1). The spread of COVID-19 cases has resulted in a worldwide pandemic with unprecedented illness and impact on society. SARS-CoV-2 is a *Betacoronavirus* and is closely related to SARS-CoV-1 and Middle East respiratory syndrome coronavirus (MERS-CoV). These three viruses all cause severe respiratory infections with high levels of pathogenicity that contribute to mortality, and a variety of morbidity (2 – 4). Up to this point, the only drugs for the treatment of COVID-19 approved fully or through emergency use authorization (EUA) by the US Food and Drug Administration include various monoclonal antibodies (5 – 7), immune modulators (8), and three antiviral drugs (9, 10).

Monoclonal antibodies specifically target the viral spike protein and block entry into cells to neutralize the SARS-CoV-2 virus. Currently, several monoclonal antibodies are authorized for use via an EUA including casirivimab and imdevimab (REGEN-COV) (5) and sotrovimab (11). Their efficacy has been evident when utilized at early stages of infection; however, the emergence of new variants (12), most notably the Omicron variant in late 2021 (13) and subvariants within the Omicron lineage emerging in 2022, has rendered many of these monoclonal antibodies largely ineffective (14). Additionally, these antibodies must be administered intravenously, making them less accessible than oral medications.

Immune modulators represent another category of treatment for COVID-19, as they can be used for COVID-19 patients to either suppress hyperinflammation and cytokine storm or to boost another aspect of the immune system to prevent the worsening of the disease. Baricitinib functions as a Janus Kinase inhibitor that is authorized under an EUA for patients receiving supplemental oxygen or mechanical ventilation (15). Tocilizumab is a monoclonal antibody that functions by blocking the interleukin-6 (IL-6) receptor for patients receiving corticosteroids and supplemental oxygen (16). Dexamethasone is a corticosteroid recommended for use in hospitalized patients with COVID-19 requiring supplemental oxygen (17). Notably, these immunosuppressants are only to be used when patients have reached a severe state of disease.

The subsets of drugs used clinically for antiviral treatment of COVID-19 are remdesivir (i.v. administered), paxlovid, and molnupiravir which are both orally administered. Remdesivir is a prodrug that works to inhibit the viral RNA polymerase via its transformation into a ribonucleotide analog inhibitor (18). Remdesivir is highly expensive and is a drug infusion given intravenously, making it a much more difficult treatment to administer on a large scale. Molnupiravir functions as a prodrug of a synthetic nucleoside derivative that introduces errors into viral RNA replication (19); however, there are drawbacks including the possibility that this drug could accelerate the emergence of variants of concern as well as host issues associated with mutagenesis (20) as well as a recent study showing much lower efficacy compared to previous reports (21). Paxlovid is composed of two drugs, the SARS-CoV-2 main protease (Mpro) inhibitor nirmatrelvir as well as ritonavir, a drug that helps inhibit cytochrome P450-3A4 preventing the normal metabolism of protease inhibitors (22). This has been proven to be the most effective treatment so far and is widely used; however, there are many issues associated with the usage of ritonavir such as the contraindication with many other commonly used drug classes. Additionally, there have been reports of the emergence of SARS-CoV-2 mutations that evade the efficacy of Paxlovid, raising concerns over the emergence of viruses adapted to this drug (13, 23). There is an urgent need for antivirals that show efficacy as a single-agent treatment, or as a combination regimen along with other approved therapeutics to address the current and future threat of continued coronavirus infections, outbreaks, and drug resistance.

Through previous collaborative studies, several drugs have been identified to interact with SARS-CoV-2 that have led to the repurposing of existing drugs such as references (24 – 26), which had initially been tested for Ebola virus and other coronaviruses. Finding existing drugs with efficacy against SARS-CoV-2 represents the fastest and most cost-effective available method for improving treatment for COVID-19 patients. Previously, pyronaridine tetraphosphate was identified as a potential SARS-CoV-2 antiviral by multiple *in silico* studies (27, 28) and then verified as an effective antiviral against SARS-CoV-2 using *in vitro* drug screens against multiple compounds with potential efficacy against SARS-CoV-2 (29). Additionally, pyronaridine tetraphosphate has previously been identified as an inhibitor of the Ebola virus and Marburg virus *in vitro*. Pyronaridine belongs to a family of anti-malarial drugs that include tilorone, quinacrine, hydroxychloroquine, and chloroquine, all have the potential to act as lysomotrophs, meaning they inhibit the acidification of endosomal compartments and can function as entry inhibitors (30).

Synthesized in 1970 as an antimalarial drug, pyronaridine’s usage has been confined to patients suffering from malaria and can be used in combination with another anti-malarial compound, artesunate in a formulation named Pyramax (31). This drug is produced cheaply and is efficacious in the treatment of malarial infections, functioning through multiple mechanisms such as inhibiting hemozoin formation, intercalating into DNA, and inhibiting topoisomerase II. Pyronaridine is also under investigation as an anticancer drug due to its numerous host-targeting mechanisms, including induction of reactive oxygen species, modulation of hypoxia (HIF-1α [Hypoxia-inducible factor 1 subunit-alpha] inducible factor downregulation), depolarization of mitochondria, and antiproliferative effects (downregulation of cyclin D1 and Ki-67) as well as immune modulatory effects such as lowering IL-6 (30, 32 – 34). More recently, pyronaridine has been found to have antiviral efficacy in multiple strains of Ebola virus and Marburg virus *in vitro,* showing potential broad activity against filoviruses through a mechanism involving entry inhibition. Pyronaridine also displayed *in vivo* efficacy by enhancing survival rate in mice and guinea pigs against Ebola virus (35).

The usage of pyronaridine in coronavirus models includes a recent SARS-CoV-2 *in vitro* infection model utilizing immortalized A549-ACE2 cells in which pyronaridine was found to inhibit replication of the virus and have an antiviral effect (27). In a recent *in vivo* study, pyronaridine has been shown to function in K18-hACE2 expressing transgenic mice infected with an isolate of SARS-CoV-2 by reducing viral load in the lungs, reducing lung pathology, and reducing pro-inflammatory cytokine levels. This was found to occur through the potential molecular mechanism of inhibiting viral PL protease activity (28). This is particularly notable as there is a strong homology in the PL protease sequences between SARS-CoV-1, MERS-CoV, and SARS-CoV-2 with 83% identical sequence and 90% similar sequence between SARS-CoV-1 and SARS-CoV-2, and 31% identical sequence and 49% similar sequence between SARS-CoV-2 and MERS-CoV (36). Herein, we describe pyronaridine, which presents antiviral and anti-inflammatory activity toward SARS-CoV-2 infections in a BALB/c mouse model, across numerous variants and models of infection, as well as demonstrate broad pan-coronavirus activity in SARS-CoV-1 and MERS-CoV mouse models of infection. This work demonstrates that pyronaridine has broad antiviral and anti-inflammatory activity and should be considered for clinical testing in humans with COVID-19.

## RESULTS

### Prophylactic pyronaridine reduces SARS-CoV-2 infection and inflammation in mice

**Fig 1:**
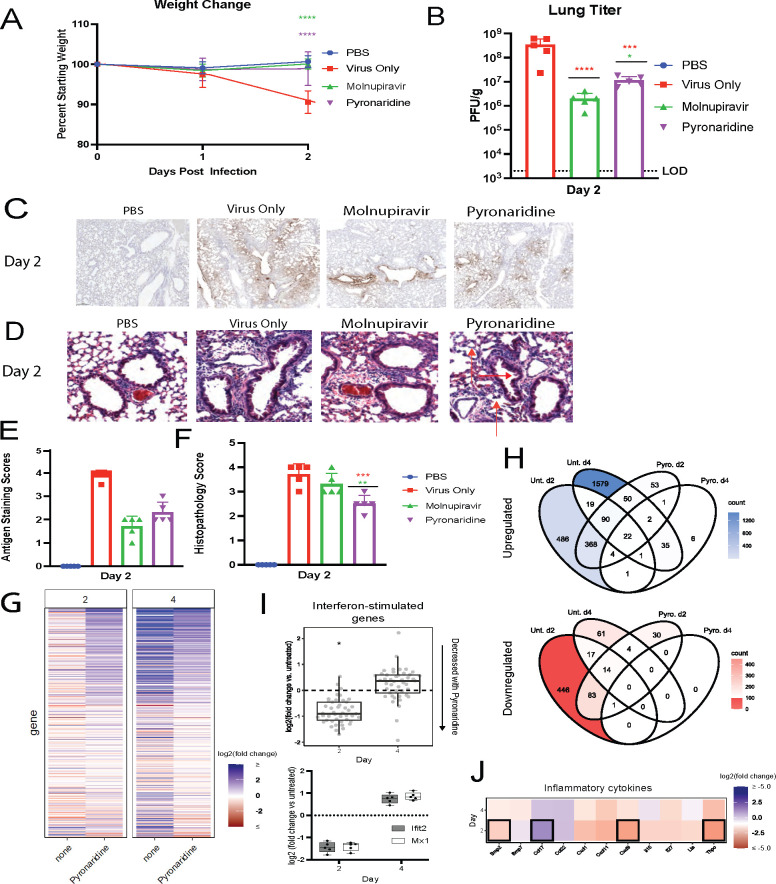
Prophylactic pyronaridine reduces SARS-CoV-2 infection and inflammation in mice. Eight- to 10-week-old wild-type Balb/c mice (*n* = 5 per group) were challenged with 1 × 10^5^ PFU of SARS-CoV-2 Beta variant (B.1.351). And treated with either nothing, molnupiravir, or pyronaridine orally beginning 12 h before infection. (**A**) Mouse weights were measured each day and lungs were harvested at 2 dpi for (**B**) viral titer quantification, (**D**) lung histology staining with red arrows indicating reduced lung inflammatory infiltrates and bronchovascular cuffing and (**F**) scoring analysis, and (**C**) nucleocapsid staining with hematoxylin and eosin and (**E**) scoring quantification for interstitial inflammation. Lungs were harvested at 2 dpi and 4 dpi for RNA extraction and RNA-sequencing analysis including (**G**) heatmap showing fold changes of genes relative to uninfected mice. Only genes differentially expressed at any time point (at least twofold expression change in either direction, adjusted *P*-value <0.01). (**H**) Venn diagrams showing sets of significantly upregulated (top, blue) or downregulated (bottom, red) genes, relative to mock, across the time points and treatments tested. Unt. = untreated. (**I**) Fold change of interferon-stimulated genes (ISGs) with pyronaridine treatment (treated vs untreated, all infected). Day 2 mean ISG levels are lower with pyronaridine treatment (*P* = 9.3e−15). (**J**) Heatmap showing the most different cytokines with pyronaridine treatment, relative to mock. Heatmap tiles outlined in black are significantly increased or decreased (adjusted *P*-value <0.01). n = 5 mice per group, mean ± SD is shown. *P < 0.05, **P < 0.01, and ***P < 0.001, using one-way analysis of variance with Dunnett’s multiple comparison test. The red asterisks are compared to virus-only group, whereas the green and purple asterisks are compared to molnupiravir and pyronaridine groups, respectively.

To study the effects of pyronaridine on SARS-CoV-2 infection, we used our previously established mouse model of SARS-CoV-2 infection. In this model, 1 × 10^5^ plaque forming units (PFU) of B.1.351 (Beta variant) are used to infect 8- to 10-week-old BALB/c mice that typically results in moderate levels of weight loss (5%–15% body weight), high viral titers around 10^8^ PFU/g in the lung by 2 days post-infection (dpi), and high levels of lung inflammatory pathology and pro-inflammatory cytokines (37). Mice were given drug treatments beginning 12 h before infection, with 100 mg/kg of pyronaridine given once per day orally and 50 mg/kg of molnupiravir given twice per day orally. Weight loss was tracked from the day of infection until 2 days post-infection. Mice dosed with only carrier lost around 10% of their body weight compared to no weight loss in mice treated with either the positive control drug molnupiravir or with pyronaridine (Fig. 1A). We examined viral replication in the lungs of infected mice at 2 dpi, observing 10^8^ PFU/g in mice infected with no treatment compared to the pyronaridine-treated group that had a 1.5-log reduction, nearly matching molnupiravir’s 2-log reduction (Fig. 1B). To analyze the antiviral impacts visually and semi-quantitatively, nucleocapsid (N) staining for SARS-CoV-2 was done using immunohistochemistry (IHC) and revealed a significant reduction in viral staining in both molnupiravir- and pyronaridine-treated mice compared to mice given no treatment (Fig. 1C and E). We examined the lungs of mice at 2 dpi using hematoxylin and eosin (H&E) staining and observed a reduction in both inflammatory infiltrates to the alveolar space as well as reduced vascular cuffing seen in mice treated with pyronaridine compared to the untreated mice; these are depicted with red arrows pointing at these features (Fig. 1D and F). Mouse lungs were also used for RNA-sequencing transcriptomic analysis at 2 dpi and 4 dpi.

From the RNAseq data, we observed less extreme global changes in gene expression in pyronaridine-treated compared to untreated mice in response to SARS-CoV-2 infection (Fig. 1G and H). On day 2, we observed significantly decreased levels of interferon-stimulated genes (ISGs) in the infected animals treated with pyronaridine compared to the untreated animals. Expression of Ifit2 and Mx1 was measured via quantitative PCR (qPCR) to validate these results (Fig. 1I). Additionally, we identified significant differences in genes encoding four immune regulatory cytokines at day 2: Ccl17, Bmp2, Cxcl19, and Thpo (Fig. 1J). Importantly, the only cytokine with increased expression was Ccl17—decreased levels of Ccl17 have been associated with severe COVID-19 (38). The observation of lower ISG expression levels at day 2 is consistent with the observed lower viral titer and altered inflammatory cytokine protein levels with pyronaridine treatment compared to untreated mice (Fig. 1B and D). Taken together, these data suggest that the pyronaridine-treated mice experience a milder ISG and inflammatory cytokine response at day 2, which is consistent with the decreased titer observed on day 2 and may explain the reduction in pathology observed in the pyronaridine-treated mice.

### A combination of molnupiravir and pyronaridine treatment additively reduces SARS-CoV-2 infection and inflammation *in vivo* compared to single drug treatment

**Fig 2:**
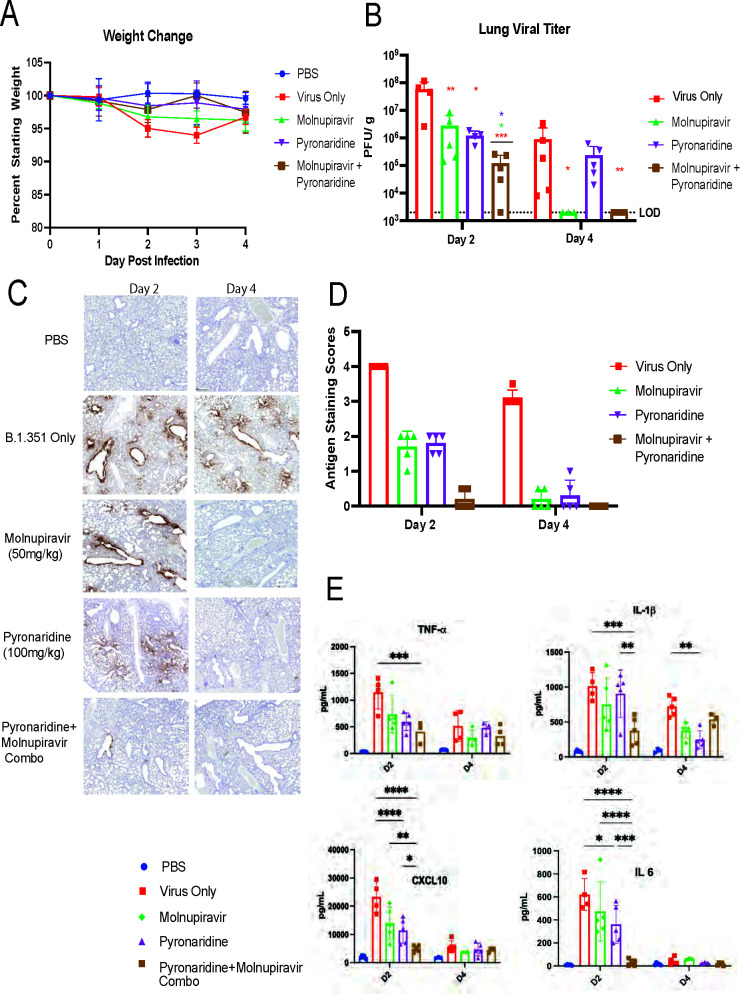
Combination of molnupiravir and pyronaridine treatment synergistically reduces SARS-CoV-2 infection and inflammation *in vivo* compared to single drug treatment. Eight- to 10-week-old wild-type BALB/c mice (*n* = 5 per group) were challenged with 1 × 10^5^ PFU of SARS-CoV-2 Beta variant (B.1.351) and treated with either nothing, molnupiravir, pyronaridine, or combination treatment of molnupiravir and pyronaridine orally beginning 12 h before infection. (**A**) Mouse weights were measured each day and lungs were harvested at 2 dpi for (**B**) mouse lungs that were analyzed for viral titer quantification by plaque assay on day 2 and day 4. (**C**) Lungs were imaged using nucleocapsid staining via IHC antibody on the lungs on day 2 and day 4 as well as by H&E staining for inflammatory pathology (Fig. S1). (**D**) Nucleocapsid staining was scored by percentage stained. (**E**) Lungs were harvested and analyzed for protein levels using a Bio-Plex Pro Mouse Chemokine Panel, a complete heat map of the chemokine/cytokine panel is available (Fig. S1). n = 5 mice per group, mean ± SD is shown. *P < 0.05, **P < 0.01, and ***P < 0.001, lung titers were analyzed for significance by log transformation and mixed-effect analysis with the Tukey test for multiple comparisons. Cytokine comparisons were analyzed by mixed-effect analysis followed by the Sidak test for multiple comparisons. The red asterisks are compared to virus-only group, whereas the green and purple asterisks are compared to molnupiravir and pyronaridine groups, respectively.

Using the same mouse model of infection as in Fig. 1, B.1.351 (Beta variant) infection of BALB/c mice, we added in combination treatment where a group of mice receives both molnupiravir and pyronaridine in combination beginning 12 h before infection. In this experiment, no significant drops were seen in body weight (Fig. 2A). We quantified a 2-log reduction in viral lung titers at 2 dpi in single drug-treated groups for both pyronaridine and molnupiravir, as seen in the first experiment, and in the combination treatment group, there was an additive effect seen with a further reduction of 3 logs that were significantly different from the single treatment groups. At 4 dpi, molnupiravir and the combination treatment were both below the limit of detection while pyronaridine alone gave only a 1-log reduction in titer (Fig. 2B). Using IHC staining for SARS-CoV-2 nucleocapsid protein, we observed a significant reduction in both single-drug treatment groups and a nearly complete reduction in staining with the combination treatments at 2 dpi. By 4 dpi, all treatment groups had largely reduced nucleocapsid staining to comparable levels, as seen both visually and through our scoring system (Fig. 2C and D). We performed a Luminex protein quantification assay to measure cytokine and chemokine levels in the lungs of infected and treated mice. We observed stark reductions in TNF-α (tumor necrosis factor alpha), IL-1β, CXCL10, and IL-6 in pyronaridine and molnupiravir combination-treated mice to a further degree than single treatments (Fig. 2E). A heatmap showing the full panel of fold changes gives a wider view of cytokines levels (Fig. S1). H&E staining was performed on these lung samples, but no significant differences were noted via observation or through scoring(Fig. S1). This experiment demonstrates that combination treatment enhances pyronaridine and molnupiravir inhibition compared to single drug administration.

### A combination of nirmatrelvir and pyronaridine treatment additively reduces SARS-CoV-2 infection and inflammation *in vivo*

**Fig 3:**
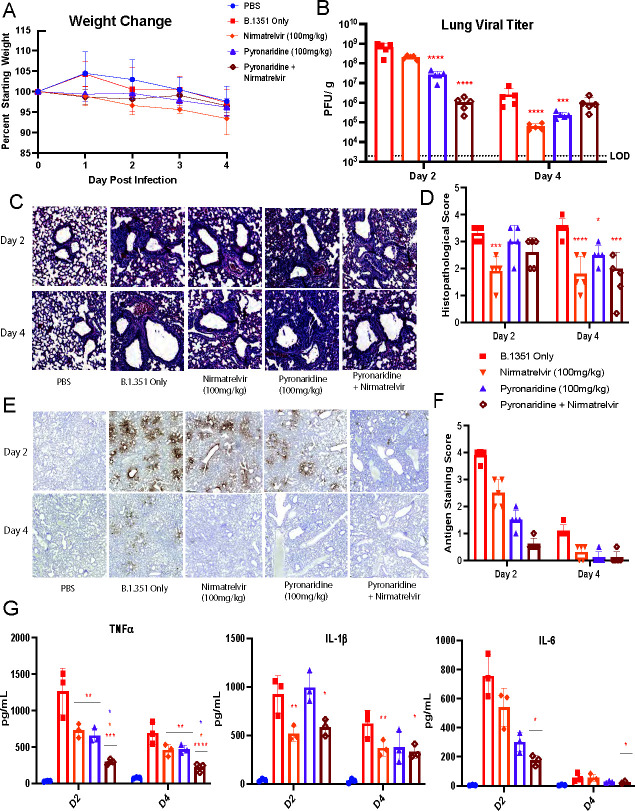
Combination of nirmatrelvir and pyronaridine treatment additively reduces SARS-CoV-2 infection and inflammation *in vivo*. Eight- to 10-week-old wild-type BALB/c mice were treated with nirmatrelvir (oral administration), and/or nirmatrelvir (oral administration) daily at the indicated concentrations starting 12 h before infection. Mice (*n* = 5 per group) were intranasally inoculated with 1 × 10^5^ PFU per mouse of the SARS-CoV-2 Beta variant (B.1.351). (**A–G**) Mice were weighed daily (**A**), lungs were analyzed for viral titer 2 and 4 days after infection by plaque assay (**B**), or fixed in 4% paraformaldehyde for H&E staining and quantified for interstitial and bronchovascular inflammation (**C and D**), or stained for SARS-CoV-2 nucleocapsid via IHC (**E**) and the staining was quantified by the percentage of tissue (**F**). Lung homogenate was also used in a Bio-Plex chemokine and cytokine protein quantification assay and select cytokines are shown (**G**), while the entire assay heatmap is shown separately(Fig. S3). n = 5 mice per group, mean ± SD is shown. *P < 0.05, **P < 0.01, and ***P < 0.001, lung titers were analyzed for significance by log transformation and mixed-effect analysis with the Tukey test for multiple comparisons. Cytokine comparisons were analyzed by mixed-effect analysis followed by the Sidak test for multiple comparisons. The red asterisks are compared to virus-only group.

Using the B.1.351 SARS-CoV-2 mouse model of infection, we tested the efficacy of oral nirmatrelvir (100 mg/kg) given twice a day beginning 12 h pre-infection, oral pyronaridine (100 mg/kg) given once per day beginning 12 h pre-infection, and a combination treatment with both therapeutics administered orally (nirmatrelvir twice per day, pyronaridine once per day). There was no weight loss observed in this experiment (Fig. 3A). Quantifying lung viral titer by plaque assay revealed no significant decreases in nirmatrelvir-treated mice at 2 dpi, whereas pyronaridine alone was reduced significantly by 2 logs, and the combination treatment was down even further with a 3 log reduction. By 4 dpi, only the single-treated mouse groups were down significantly, and the combination group was not down (Fig. 3B). Through H&E staining and quantification, it was noted that only the nirmatrelvir group at 2 dpi displayed reduced levels of lung inflammatory pathology to a significant extent, whereas at 4 dpi, both the combination treatment group and the nirmatrelvir group were down (Fig. 3C and D). Utilizing SARS-CoV-2 nucleocapsid staining on the lungs, by 2 dpi, we observed a clear reduction in the pyronaridine group and the combination group, with the nirmatrelvir group showing reduced levels of nucleocapsid to a lesser degree. By 4 dpi, the infected-only group had relatively low levels of nucleocapsid, so the reduction was visible across all treatment groups but harder to differentiate between (Fig. 3E and F). We performed a Luminex protein quantification assay to measure cytokine and chemokine levels in the lungs of infected and treated mice and observed large reductions in TNF-α, IL-1β, and IL-6 upon treatment with single drugs and further reductions in TNF-α and IL-6 for the combination treatment showing an additive effect (Fig. 3G).

We also performed a study using therapeutic dosing in this model. We infected mice intranasally, and on day 1 following infection, we treated mice with molnupiravir, nirmatrelvir, pyronaridine, or the combination of pyronaridine and molnupiravir or nirmatrelvir, and quantified viral infection and inflammation in the lungs on days 2 and 4 after infection. We found that the addition of pyronaridine to molnupiravir and nirmatrelvir further reduced viral titers and inflammation (Fig. S2). A Luminex assay was performed on this therapeutic dosing experiment and the heatmap for all proteins included shows general reductions in pro-inflammatory cytokines across single treatments with some additive reductions in combined treatments (Fig. S3).

### Therapeutic pyronaridine protects against lethal infection by mouse-adapted SARS-CoV-2 in old mice

**Fig 4:**
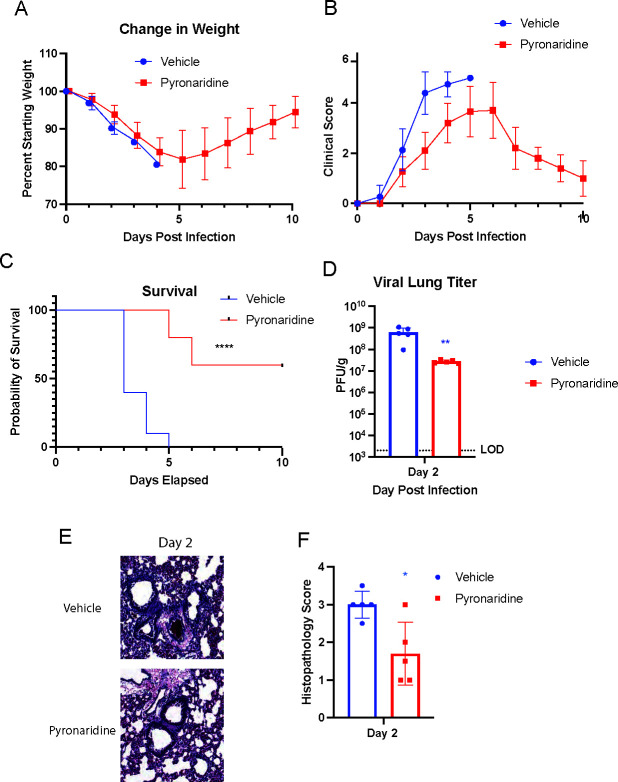
Therapeutic pyronaridine protects against lethal infection by mouse-adapted SARS-CoV-2 in old mice. Six-month-old wild-type BALB/c mice were treated with pyronaridine(oral administration), or a phosphate-buffered saline vehicle control daily at the indicated concentrations starting 1 h post-infection. Mice (*n* = 5 per group) were intranasally inoculated with 1 × 10^4^ PFU per mouse of mouse-adapted SARS-CoV-2 (MA-10). (**A–F**) Mice were weighed daily (**A**), clinical scores for disease severity were recorded daily (**B**), lethality or survival was recorded daily (**C**), lungs were analyzed for viral titer 2 days after infection by plaque assay (**D**), or fixed in 4% paraformaldehyde for H&E staining and quantified for interstitial and bronchovascular inflammation (**E and F**). Mean ± SD is shown. *P < 0.05, **P < 0.01, and ***P < 0.001, with blue asterisks indicating differences with the vehicle control group. Lung titers were analyzed for significance by log transformation and paired *t-test* analysis. A comparison of survival curves was done with log-rank (Mantel-Cox) test.

To evaluate the efficacy of pyronaridine in a severe lethal model of infection, we used 6-month-old BALB/c mice and infected them with 10^4^ PFU of mouse-adapted SARS-CoV-2 MA10 (39). Mice were treated 1 h post-infection with either a vehicle treatment (saline) or 100 mg/kg of pyronaridine once per day. The mice were weighed and given a clinical score daily until 10 dpi, and the lungs were evaluated at 2 dpi. In mice given a vehicle treatment, they began losing weight at a slightly accelerated rate compared to the pyronaridine-treated group (Fig. 4A). The daily clinical score assessment also showed accelerated severity of disease in the vehicle group compared to the pyronaridine-treated mice (Fig. 4B). The first lethality was observed at 3 dpi for vehicle-treated mice and 5 dpi for pyronaridine-treated mice, with all vehicle mice succumbing to infection by 5 dpi while 6 out of 10 pyronaridine-treated mice survived the infection, representing a large reduction in lethality (Fig. 4C). Mouse lungs that were taken at 2 dpi were analyzed for viral titer with an average of 10^9^ PFU in the vehicle-treated mice and 10^7^ PFU in the pyronaridine-treated mice, showing a clear antiviral impact with a 2 log reduction (Fig. 4D). Lungs were stained by H&E and visually the pyronaridine-treated mice showed much less vascular thickening and alveolar infiltrate compared to the vehicle-treated group (Fig. 4E), the semi-quantitative histology scoring confirmed this with a lower score in the pyronaridine-treated mice (Fig. 4F). Taken together, these data indicate pyronaridine treatment is effective at reducing the risk of lethality for mice and lowering the severity of disease, overall viral titers, and lung inflammatory pathology post-SARS-CoV-2 infection.

### Pyronaridine reduces infection against multiple omicron subvariants of SARS-CoV-2

**Fig 5:**
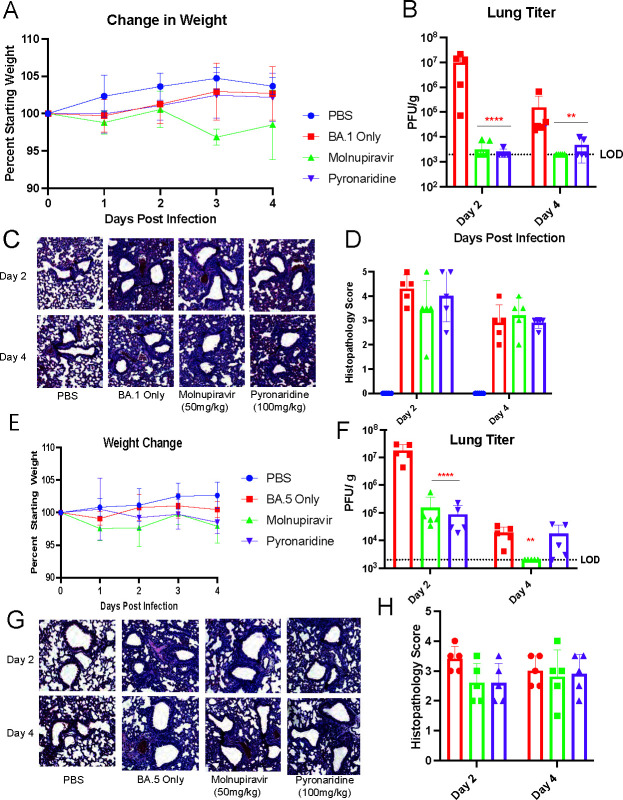
Pyronaridine reduces infection against multiple Omicron subvariants of SARS-CoV-2. Wild-type 8- to 10-week-old BALB/c mice were treated with molnupiravir (oral administration) or pyronaridine(oral administration) daily at the indicated concentrations starting 12 h before infection. Mice (*n* = 5 per group over two independent experiments) were intranasally inoculated with 1 × 10^5^ PFU per mouse of SARS-CoV-2 Omicron (BA.1 for **A–D**, and BA.5 for **E–H**). Mice were weighed daily (**A and E**), and lungs were analyzed for viral titer 2 and 4 days after infection by plaque assay (**B and F**), or fixed in 4% paraformaldehyde for H&E staining (**C and G**) and quantified for lung inflammation (**D and H**). *n* = 5 mice per group. Mean ± SD is shown. *P < 0.05, **P < 0.01, and ***P < 0.001, using two-way analysis of variance with Tukey’s multiple comparison test. The red asterisks are compared to virus-only group.

To evaluate the efficacy of pyronaridine against the Omicron variant of SARS-CoV-2, we did two separate experiments where mice were challenged with 10^5^ PFU of BA.1 or BA.5, the subvariants of Omicron that have been dominant in the United States during the year 2022. Mice were treated with either 50 mg/kg oral molnupiravir twice per day as a control, or 100 mg/kg oral pyronaridine once per day with treatments beginning 12 h before infection. Mice were weighed daily, and lungs were taken at 2 dpi and 4 dpi to be evaluated for viral titer and histopathology. Consistent with other reported results, mice infected with BA.1 had no weight loss (Fig. 5A). Control-treated mice infected with BA.1 had an average lung viral titer of 10^7^ PFU/g and 10^5^ PFU/g at 2 dpi and 4 dpi, respectively. At 2 dpi, both drug treatments dropped the viral titer by 4 logs to the limit of detection, and at 4 dpi, both drug treatments were at or near the limit of detection as well which represents about a 2 log drop in titer compared to the control group (Fig. 5B). Lungs were stained by H&E and, by appearance alone, the drug-treated groups looked slightly improved with lower levels of thickening around the vascular bundles (Fig. 5C), but the semi-quantitative scoring showed no significant reductions in any groups (Fig. 5D). Lungs were stained for SARS-CoV-2 N protein, and no detectable N protein was seen in either pyronaridine- or molnupiravir-treated lungs, whereas BA.1 only showed some detectable N protein(Fig. S4A and B), albeit much lower than in the B.1.351 infection model. Next, we challenged mice with 10^5^ PFU of BA.5, and once again no weight loss was observed (Fig. 5E). When lungs were taken for quantification of the virus, both treatment groups dropped around 2 logs from 10^7^ PFU/g to 10^5^ PFU/g at 2 dpi. At 4 dpi, only the molnupiravir group was reduced to the limit of detection, whereas the pyronaridine group showed no significant reduction (Fig. 5F). Lungs were stained by H&E and there was significant inflammation visible at both 2 dpi and 4 dpi (Fig. 5G), with both treatment groups looking slightly improved with less cell infiltrate in the alveoli. This was evident in the scoring at 2 dpi where both treatment groups had slightly reduced histopathology scores, but no differences were obvious at 4 dpi (Fig. 5H). We performed a Luminex protein quantification assay to measure cytokine and chemokine levels in the lungs of infected and treated mice and observed less overall pro-inflammatory cytokine induction compared to previous Beta variant challenges but still observed large reductions in TNF-α, IL-1β, and IL-6 upon treatment with single drugs in either BA.1 challenge (Fig. S4C) or BA.5 challenge(Fig. S4D).

### Pyronaridine reduces infection against mouse-adapted SARS-CoV-1 *in vivo*

**Fig 6:**
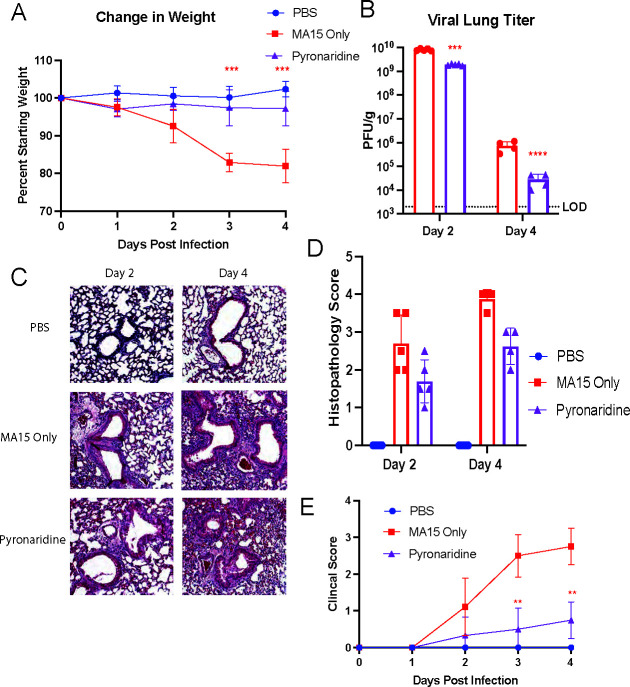
Pyronaridine reduces infection against mouse-adapted SARS-CoV-1 *in vivo*. Wild type 8- to 10-week-old BALB/c mice were treated with pyronaridine (oral administration) daily at the indicated concentrations starting 12 h before infection. Mice (*n* = 5 per group) were intranasally inoculated with 1 × 10^5^ PFU per mouse of SARS-CoV-1 mouse-adapted strain (MA-15). (**A–E**) Mice were weighed daily (**A**), lungs were analyzed for viral titer 2 and 4 days after infection by plaque assay (**B**), or fixed in 4% paraformaldehyde for H&E staining (**C**) and quantified for lung inflammation scoring (**D**). Mice were monitored and given a clinical score for disease severity daily (**E**). *n* = 5 mice per group. Mean ± SD is shown. *P < 0.05, **P < 0.01, and ***P < 0.001, using two-way analysis of variance with Tukey’s multiple comparison tests on log-transformed virus titers. The red asterisks are compared to virus-only group.

SARS-CoV-1 and SARS-CoV-2 share a high degree of homology and similarity in their papain-like protease with 83% of the sequence remaining identical and 90% similar between the two overall (36). Pyronaridine has been shown to inhibit SARS-CoV-2 PLpro (28); therefore, we examined pyronaridine’s efficacy against mouse-adapted SARS-CoV-1 MA15 in a mouse model as previously published (40). We infected 8- to 10-week-old BALB/c mice with 10^4^ PFU of SARS-CoV-1 MA15 and monitored weight loss and clinical scores daily. At 2 dpi and 4 dpi, lungs were harvested for histology and viral titer quantification. Mice were either untreated or given daily oral doses of 100 mg/kg pyronaridine beginning 12 h before infection. Mice were weighed and those infected and untreated lost around 20% body weight by 4 dpi, whereas treated mice had a strikingly better phenotype with no weight loss (Fig. 6A). Through recording clinical scores each day, we found that mice given the virus only were noticeably worse by 2 dpi, and significantly worse when compared to pyronaridine-treated mice at 3 dpi and 4 dpi (Fig. 6E). Lungs were removed at 2 dpi and viral titer quantified; we found close to 10^10^ PFU/g by 2 dpi and 10^6^ PFU/g by 4 dpi in the MA15-only group. In the pyronaridine-treated group, we found a significant reduction in viral titer with a 1-log reduction by 2 dpi and around a 2 log reduction by 4 dpi (Fig. 6B). Lungs were also embedded, sectioned, and stained using H&E to examine the lung histopathology; the MA15 virus-only group displayed significantly more inflammation with dramatic thickening around the bronchioles and increased infiltrate of immune cells in the alveolar spaces. The pyronaridine-treated group showed a reduction in these metrics visually (Fig. 6C) as well as a clear reduction in the semi-quantitative scoring of these lungs (Fig. 6D). Taken together, this experiment demonstrates that pyronaridine is an efficacious antiviral for SARS-CoV-1 infection in the mouse model that additionally improves the severity of the disease as well as the lung inflammatory pathology observed. This efficacy toward SARS-CoV-1, in addition to the SARS-CoV-2 infection data, makes this a promising therapeutic across multiple highly pathogenic coronaviruses.

### Pyronaridine reduces infection against mouse-adapted MERS-CoV *in vivo*

**Fig 7:**
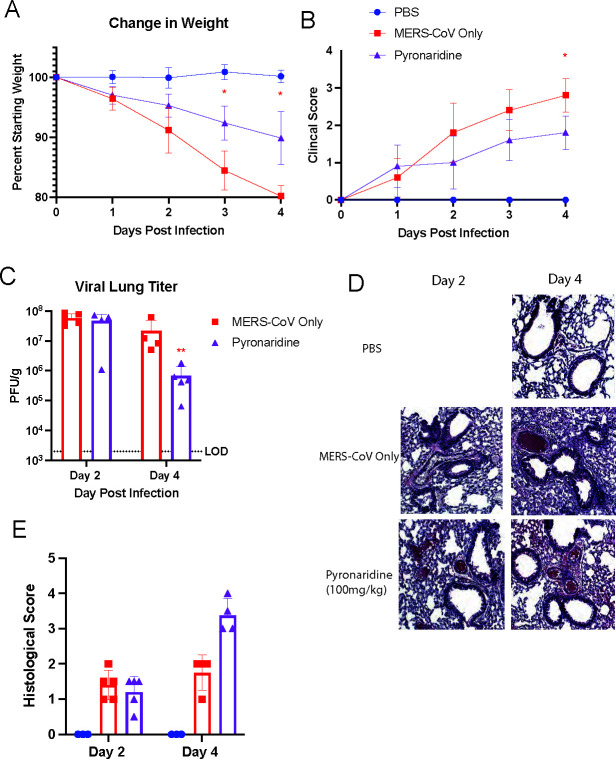
Pyronaridine reduces infection against mouse-adapted MERS-CoV *in vivo*. hDPP4 knock-in mice aged 10–12 weeks were treated with pyronaridine (oral administration) daily at 100 mg/kg starting 12 h before infection. Mice (*n* = 5 per group) were intranasally inoculated with 5 × 10^3^ PFU per mouse of MERS-CoV mouse-adapted strain. (**A–E**) Mice were weighed daily (**A**), mice were monitored and given a clinical score for disease severity daily (**B**), lungs were analyzed for viral titer 2 and 4 days after infection by plaque assay (**C**), or fixed in 4% paraformaldehyde for H&E staining (**D**) and quantified for lung inflammation scoring (**E**). n = 5 mice per group, mean ± SD is shown. *P < 0.05, **P < 0.01, and ***P < 0.001, red asterisks indicate comparisons with virus-only group. Lung titers were analyzed for significance by log transformation and mixed-effect analysis with the Tukey test for multiple comparisons.

SARS-CoV-1, MERS-CoV, and SARS-CoV-2 all share homology in their PL protease that pyronaridine is known to inhibit, with MERS-CoV being the less homologous of the three with 31% identical sequence and 49% similar sequence compared to SARS-CoV-2 (36). Therefore, we sought to investigate pyronaridine’s efficacy against MERS-CoV using a mouse model of infection. To do this, we utilized humanized DPP4 (hDPP4) receptor knock-in mice and a mouse-adapted MERS-CoV virus as reported previously (41). We infected 8- to 10-week-old hDPP4 mice with 5 × 10^3^ PFU of mouse-adapted MERS-CoV, as this inoculum had been reported to cause severe disease in these mice for a clear phenotype and then treated mice with an oral daily dose of 100 mg/kg of pyronaridine beginning 12 h before infection. Mice were weighed daily, clinical scores were recorded, and lungs were evaluated for viral load and pathology at 2 dpi and 4 dpi. After infection, mice lost up to 20% of their body weight by 4 dpi, whereas mice treated with pyronaridine only lost an average of 10% body weight (Fig. 7A). Clinical scoring also confirmed that the disease was less severe in the pyronaridine-treated group from 2 dpi to 4 dpi (Fig. 7B). Lungs were taken for viral titering, and it was found that at 4 dpi, mice treated with pyronaridine had significantly lower viral load with around a 2 log drop from 10^7^ PFU/g to 10^5^ PFU/g (Fig. 7C). Lungs were also stained by H&E to compare lung inflammatory pathology (Fig. 7D), and visually, there were no obvious differences by 2 dpi between the treated and untreated mice. By 4 dpi, the scoring system showed the pyronaridine-treated group had an increased histopathology score on average (Fig. 7E). This appears to be a different readout than in SARS-CoV-2 and SARS-CoV-1 infections where inflammatory pathology was reduced with pyronaridine treatment in many experiments. We analyzed a panel of chemokines and cytokines using a Luminex assay and found that many were not dramatically altered between the MERS-CoV infection only and the pyronaridine-treated group (data not shown) indicating a potentially direct antiviral mechanism with no obvious anti-inflammatory effects.

## DISCUSSION

SARS-CoV-2 infections and COVID-19 continue to be one of the largest current threats to human health. With limited options of medical countermeasures available due to variant evolution reducing the effectiveness of monoclonal antibodies, currently approved medications such as Paxlovid and molnupiravir are the only direct-acting antiviral treatment options. Pyronaridine represents a hugely promising therapeutic against multiple coronavirus infections due to its low cost of production, oral administration, and proven safety record in malaria treatment in Africa and Asia (42). Previously, pyronaridine has been shown to have efficacy in cellular *in vitro* models as well as in one K18-hACE2 mouse model experiment (27, 28). In our current study, we utilized a BALB/c mouse model for analyzing the efficacy of pyronaridine as an orally administered drug against a variety of SARS-CoV-2 variants, and the other two highly pathogenic human coronaviruses, SARS-CoV-1 and MERS-CoV.

We find that pyronaridine is efficacious as both an antiviral and anti-inflammatory treatment when used prophylactically or therapeutically in mice given an exceedingly pathogenic, high dose of the SARS-CoV-2 Beta variant. In this model, we found a significant reduction in viral lung titers following the administration of pyronaridine as compared to untreated mice, and comparable antiviral effects to other clinically proven drugs such as molnupiravir and nirmatrelvir (the protease inhibitor component of Paxlovid). Additionally, we see reductions in lung inflammatory pathology, SARS-CoV-2 nucleocapsid levels by immunohistochemical labeling, interferon-stimulated genes induction, and a panel of inflammatory cytokines and chemokines associated with severe COVID-19 and lethal disease in humans.

We next tested the efficacy of combination treatments of pyronaridine with clinically available antivirals such as molnupiravir and nirmatrelvir to determine whether combination therapy could be more efficacious than single drug treatment alone. We find that the combination treatment of pyronaridine with molnupiravir has a synergistic antiviral effect, reducing viral titers and SARS-CoV-2 nucleocapsid levels in the lungs beyond these levels found in individual treatments. Additionally, we find this combination has larger reductions in inflammatory cytokines associated with “cytokine storm.” Evaluating pyronaridine in combination with nirmatrelvir produces similar results, with a clear additive antiviral effect of both drugs seen in nucleocapsid staining in the lungs, as well as a significant reduction in inflammatory cytokines and chemokines post-infection. These results indicate pyronaridine’s potential as a future combinable treatment for COVID-19 patients.

To challenge the efficacy of pyronaridine in a more lethal model, we tested whether pyronaridine treatment following infection using a mouse-adapted SARS-CoV-2 MA-10 virus is sufficient to prevent lethality in mice (43). Whereas 100% of the untreated mice died, mice given pyronaridine once per day following infection survived at a rate of 60%. Along with this dramatically improved survival, there was also a clear reduction in weight loss, clinical disease score, and lung viral titer in these treated mice. This shows the potential of pyronaridine in even the most severe COVID-19 disease manifestations.

As SARS-CoV-2 variants have emerged, mutations throughout the viral genome have been identified with critical mutations in the spike protein being important for vaccine and antibody design. The predicted direct-acting antiviral target of pyronaridine is the papain-like protease (27). Mutations in other proteins in the variants could have functional consequences on viral replication kinetics, entry, and protease activity such that testing was warranted against current Omicron variants *in vivo.* We find that pyronaridine treatment against multiple subvariants of the Omicron variant (BA.1 and BA.5) is highly effective as an antiviral treatment with the largest reduction in lung viral titer we see in any of our experiments. Based on these data, pyronaridine may be even more efficacious against Omicron infections than against more highly pathogenic variants like the Beta variant, which is now extinct.

The ability to inhibit more broadly against coronaviruses is critical for the development of therapeutics in the future. Inhibiting SARS-CoV-2 produces a therapeutic that is useful now but our goal is to identify a therapeutic that is effective against those coronaviruses we know of and those that we have not yet identified (44, 45). To do this, we tested the effects of pyronaridine in highly pathogenic mouse models of SARS-CoV-1 and MERS-CoV. The challenge model for each provides a high bar for protection against viral infection, lung titer, and histopathology. The PLpro of coronaviruses is conserved at the active site, suggesting that a drug that targets this site may have broad antiviral effects (46, 47). We find that pyronaridine treatment is highly effective as an antiviral in SARS-CoV-1 and MERS-CoV infections. We observed reductions in viral titer upon treatment compared to untreated mice as well as greatly improved clinical scores, weight levels, and levels of lung inflammatory pathology. While these mechanisms of antiviral impact may differ, it is likely that the drug is targeting the homologous papain-like protease that all these coronaviruses utilize as has been reported previously.

The emergence of SARS-CoV-2 variants has reduced the availability of usable therapeutics. The loss of monoclonal antibody therapies due to spike mutations for clinical care especially in highly susceptible patients will lead to increased use of Paxlovid, a SARS-CoV-2 main protease inhibitor. The active site of Mpro is known and increasing numbers of SARS-CoV-2 mutant viruses that are resistant to Paxlovid are being identified (13, 23). The essentiality of both PLpro and Mpro toward coronavirus replication makes them ideal targets for antiviral development. Combination therapy of protease inhibitors that target both proteases would provide a limited chance of escape generation compared to monotherapy, especially for a fast-replicating virus. Combination of a protease inhibitor with a replication inhibitor such as molnupiravir would also provide inhibition of multiple aspects of the coronavirus replication cycle. As we have shown here, combination therapy *in vivo* is highly effective and better than either single drug alone. In addition, our studies show that there are potent anti-inflammatory effects of pyronaridine in our mouse models that are not seen for either molnupiravir or nirmatrelvir, which may enhance its therapeutic efficacy. Finally, the oral dosing of pyronaridine and the lack of a required co-drug, like ritonavir, make it easily prescribed and taken by patients.

We propose that pyronaridine represents a highly promising therapeutic against SARS-CoV-1, MERS-CoV, and all variants of SARS-CoV-2 as well as a promising combination treatment with currently approved drugs such as molnupiravir and nirmatrelvir. Pyronaridine is on the list of WHO essential medicines, is widely used for anti-malarial treatments, and is under study for its antiviral impacts on other viruses. We are currently evaluating the potential other mechanisms of pyronaridine antiviral efficacy beyond protease inhibition, as well as exploring creating analog compounds that have improved levels of inhibition on coronaviruses.

## MATERIALS AND METHODS

### Viruses and cells

Cells and viruses were handled and processed as published previously (48). Vero-E6 cells (ATCC# CRL 1586) were cultured in DMEM (Quality Biological), with 10% (vol/vol) FBS (Gibco), 1% (vol/vol) penicillin/streptomycin (Gemini Bio-Products), and 1% (vol/vol) L-glutamine (Gibco). Cells were grown at 37°C with 5% CO_2_. The SARS-CoV-2 Beta variant was provided by A. Pekosz (Johns Hopkins University School of Public Health). SARS-CoV-2 Omicron subvariant was provided by M. Suthar (Emory University Vaccine Center), and the mouse-adapted SARS-CoV-2 MA10 strain was provided by R. Baric (University of North Carolina Gillings School of Public Health). Virus stocks were amplified using the ARTIC primer set and sequenced using the MinION system (Oxford Nanopore Technologies) by the J. Craig Venter Institute (MD, USA) to more than 4,000× genome coverage. The stock sequence was verified by aligning reads to the reference genome provided by the BEI (Beta variant GISAID accession: EPI_ISL_890360, Omicron variant GISAID accession: EPL_ISL_7171744) using minimap2 version 2.22 with the ‘map-ont’ presets, followed by inspection of the consensus sequence and alignment using IGV (Integrative Genomics Viewer).

Stocks had a less than 1% variation. Media were collected and clarified by centrifugation before being aliquoted for storage at −80°C. Titer of stock was determined by plaque assay using Vero-E6 cells expressing TMPRSS2 as previously described (49). All work with infectious viruses was performed in a biosafety level 3 laboratory and approved by the University of Maryland School of Medicine Institutional Biosafety Committee.

### Animal studies

The University of Maryland School of Medicine is accredited by the Association for Assessment and Accreditation of Laboratory Animal Care (AAALAC International). All animal procedures were done in accordance with the NRC (National Research Council) Guide for the Care and Use of Laboratory Animals, the NIH (National Institutes of Health)/CDC (Centers for Disease Control and Prevention) Biosafety Guidelines in Microbiological and Biomedical Laboratories, and the Animal Welfare Act. All mouse studies were approved by the University of Maryland School of Medicine Institute for Animal Care and Use Committee. Studies were done in accordance with the NIH Health Guide for Care and Use of Laboratory Animals (NIH publication 8023, revised 1978). All mouse infections were carried out in an animal biosafety level 3 facility in accordance with pre-approved practices.

### Mouse infections

Eight- to 10-week-old female BALB/c and 6-month-old BALB/c mice were purchased from Jackson Laboratories. On day 0, mice were given an intraperitoneal injection of 50 μL mix of ketamine (1.3 mg/mouse) and xylazine (0.38 mg/mouse) diluted together in phosphate-buffered saline (PBS). While anesthetized, mice were intranasally inoculated with either 50 μL of sterile PBS or virus. The viruses used were B.1.351 Beta variant of SARS-CoV-2, mouse-adapted SARS-CoV-2 MA-10 , BA.1 and BA.5 Omicron variants of SARS-CoV-2, or mouse-adapted SARS-CoV-1 MA-15. Humanized DPP4 knock-in mice were graciously given by the University of Iowa Perlman laboratory and used for the MERS-CoV infection in the same manner as described above. Mice were monitored daily for weight loss and signs of morbidity. Mice were euthanized on days 2 and 4 following infection with lung tissue collected for analysis.

### Drug administration

Mice were given an oral gavage volume of 100 μL per drug administration. For molnupiravir (#HY-135853, MedChemExpress), the drug was prepared in 10% DMSO (dimethyl sulfoxide) (2438, Sigma) and 90% corn oil (8627, Sigma), and administered twice daily. For nirmatrelvir (HY-138687, MedChemExpress), the drug was prepared in 10% DMSO and 90% corn oil, and administered twice daily. Pyronaridine (HY-14749A, MedChemExpress) was prepared in 10% DMSO and 90% saline, and administered once daily. For prophylactic dosing, drugs were administered beginning 12 h before infection. For therapeutic dosing, drugs were administered either 1 h or 24 h following infection.

### Histology and immunohistochemistry

Mouse lungs were fixed in 4% paraformaldehyde (PFA) in PBS for at least 48 h. These lungs were then sent to the University of Maryland Baltimore Histology core facility for paraffin embedding, sectioning into 5 μm sections, and staining with hematoxylin and eosin staining.

Lungs were scored in a blinded fashion with a 0 to 5 score given, 0 being no inflammation and 5 being the highest degree of inflammation. Interstitial inflammation and peribronchiolar inflammation were scored separately. Scores were then averaged for the overall inflammation score. For SARS-CoV-2 nucleocapsid immunohistochemistry staining, lung samples were similarly fixed in 4% PFA in PBS solution for 48 h; they were then embedded into paraffin. Paraffin blocks were sent to Histowiz (Brooklyn, NY) for staining. Paraffin blocks were sectioned and immunostained using a monoclonal antibody for SARS-CoV-2 nucleocapsid (GTX635686). IHC was scored based on the percentage distribution of staining in the lung tissue with every number representing 25%: 0 = no staining, 1 = 0%–25%, 2 = 25%–50%, 3 = 50%–75%, 4 = 75%–100% staining of tissue.

### Virus titers/plaque assay

Vero-E6/TMPRSS2 cells (50) were cultured in DMEM (Dulbecco’s Modified Eagle Medium) (Quality Biological), supplemented with 10% FBS (Sigma), 1% (vol/vol) penicillin/streptomycin (Gemini Bio-products), and 1% (vol/vol) L-glutamine (Gibco). Cells were grown and maintained in an incubator at 37°C and 5% CO_2_. Viral lung titers were quantified by homogenizing lung tissues in PBS (Quality Biological) with 1.0 mm glass beads (Sigma) in a Beadraptor (Omni International). Vero-E6 cells were plated in 12-well plates with 2.0 × 10^5^ cells per well. Plaque assay was performed by adding 25 μL lung homogenate after centrifugation to 225 μL DMEM with 10-fold dilutions across a six-point dilution curve with 200 μL DMEM diluent added to each of the wells. After a 1-h dilution with plate rocking every 15 min, a 2 mL agar overlay containing DMEM is added to each of the wells. Plates are incubated for 2 days for all viruses except Omicron subvariants (3 days) at 37°C and 5% CO_2_. Then plaques are fixed with 10% formalin, stained with crystal violet, washed with tap water, and counted.

### Clinical scoring

Clinical signs of disease were assessed daily in mice. Clinical scores were determined on the following scale as previously reported (51): 0 = healthy; 1 = slight ruffling of the fur, altered hind limb posture; 2 = mildly labored breathing, no lethargy, 3 = moderately labored breathing, lethargy; 4 = severely labored breathing, severe lethargy; and 5 = dead.

### Cytokine and chemokine quantification

To quantify the immune response induced by SARS-CoV-2 infection in mice, lung tissue was homogenized and a Bio-Plex Pro Mouse Chemokine Panel 31-plex (BIORAD) was used to quantify an array of cytokines and chemokines as listed in the manufacturer protocol. An additional fixation step is added at the end of the protocol following the last wash. Samples are fixed in 4% formaldehyde (Sigma) at 4°C. The plate is then washed three more times in wash buffer and analyzed on a Luminex MagPix and xPONENT Software version 4.3. Using a standard curve, concentrations of each analyte are calculated for all samples.

### RNA extraction

Mouse lung tissue was homogenized in 1 mL of TRIzol (Ambion) using 1.4 mm glass beads (Omni International) and a Beadruptor (Omni International). RNA was extracted using the DIRECT-zol mini-RNA Extraction kit (Zymo Research) per the manufacturer’s instructions.

### RNA-sequencing

After RNA extraction, library preparation and sequencing were performed by the University of Maryland Institute of Genome Sciences (Baltimore, MD, USA) on an Illumina NovaSeq 6000 (S4 flow cell, 100 bp paired-end; Illumina, San Diego, CA, USA). The raw reads are available in the NCBI Sequence Read Archive under the accession number PRJNA984149. Raw data were preprocessed with cutadapt v3.4 (52) and aligned to the murine genome (assembly GRCm38) using the STAR aligner v2.7.8a (53). Differential expression analysis was performed using DESeq2 v4.1.0 (54) in R (R studio, Boston, MA, USA); subsequent pathway analysis was performed using Ingenuity Pathway Analysis (QIAGEN, Hilden, Germany). We selected an alpha value of *P*_adj_ < 0.01 to call significantly differentially expressed genes.

### Statistical analyses

Statistics were performed with GraphPad Prism 9.3.1 software (GraphPad Software, San Diego, CA). Data were analyzed using unpaired *t-test*, one-way or two-way analysis of variance (ANOVA) followed by Tukey, Dunnett, or Sidak post hoc comparison test as indicated. All statistical analyses were two-sided and a *P* < 0.05 was considered statistically significant. For the survival study, the Kaplan-Meier survival curve was used, and log-rank (Mantel-Cox) test was performed to determine significance. For RNA-seq analysis, one-way ANOVA followed by *t*-test with Bonferroni correction was used, where indicated. For the differential expression analysis, reported *P*-values are the result of the Wald test after Benjamini-Hochberg correction, as calculated by DESeq2 v4.1.0 (54). Statistical methods were not used to predetermine the sample size. Blinding and randomization were not used.

## SUPPLEMENTAL MATERIAL

Fig. S1 mbio.01587-23-s0001.tif Related to Fig. 2.

Fig. S1 legend mbio.01587-23-s0002.tif Legend to Fig. S1.

Fig. S2 mbio.01587-23-s0003.tif Related to Fig. 2 and 3.

Fig. S2 legend mbio.01587-23-s0004.tif Legend to Fig. S2.

Fig. S3 mbio.01587-23-s0005.tif Related to Fig. 2 and 3.

Fig S3 legend mbio.01587-23-s0006.tif Legend to Fig. S3.

Fig. S4 mbio.01587-23-s0007.tif Related to Fig. 5.

Fig. S4 legend mbio.01587-23-s0008.tif Legend to Fig. S4.

ASM does not own the copyrights to Supplemental Material that may be linked to, or accessed through, an article. The authors have granted ASM a non-exclusive, world-wide license to publish the Supplemental Material files. Please contact the corresponding author directly for reuse.
