# Supplementary figures and images for: Pyronaridine tetraphosphate is an efficacious antiviral and anti-inflammatory active against multiple highly pathogenic coronaviruses
Source: mBio. 2023 Aug 15;14(5):e01587-23. doi: 10.1128/mbio.01587-23 (PMC10653794; doi:10.1128/mbio.01587-23)

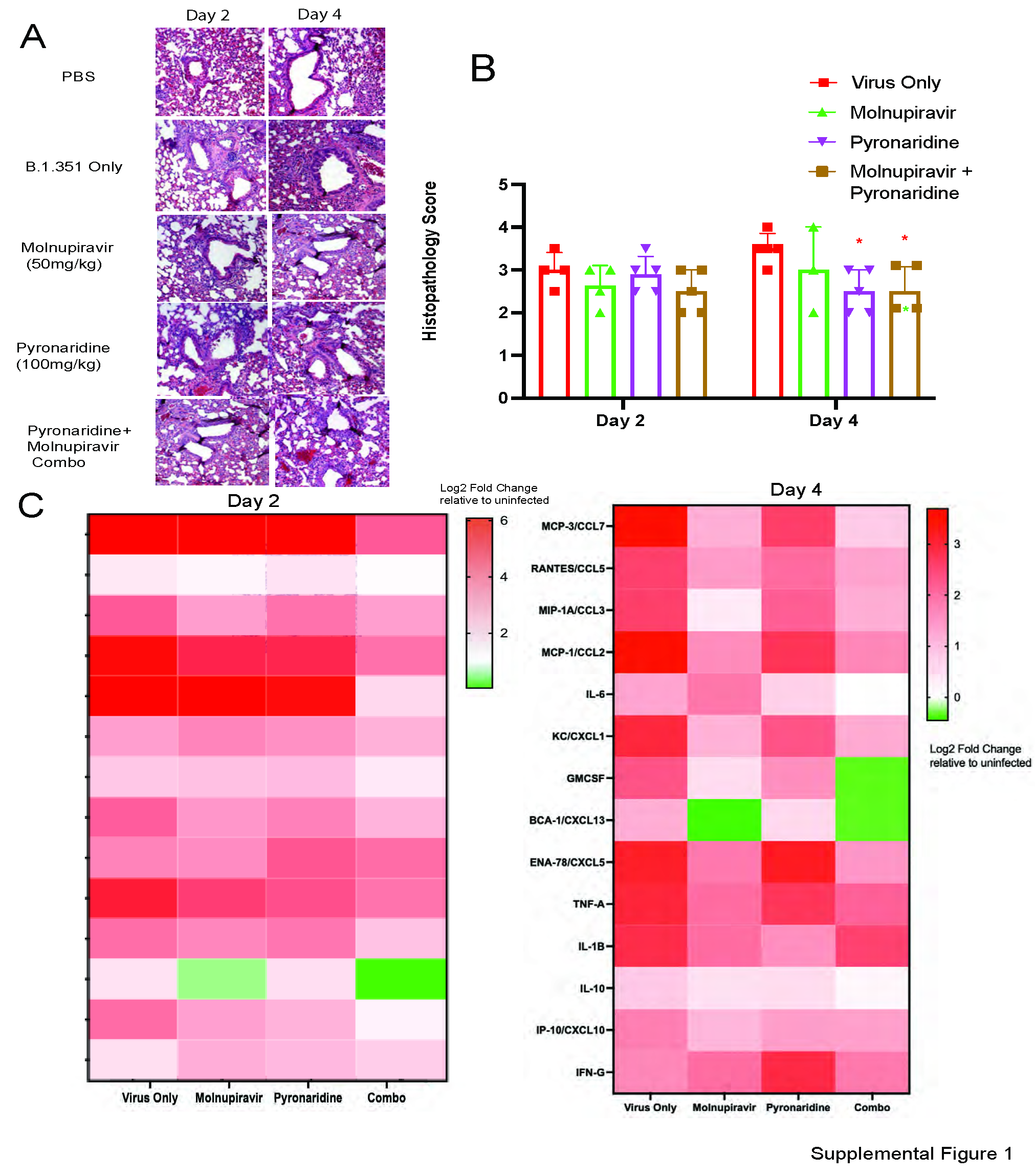

Supplement: Fig. S1 — Related to Fig. 2. [file mbio.01587-23-s0001.tif]

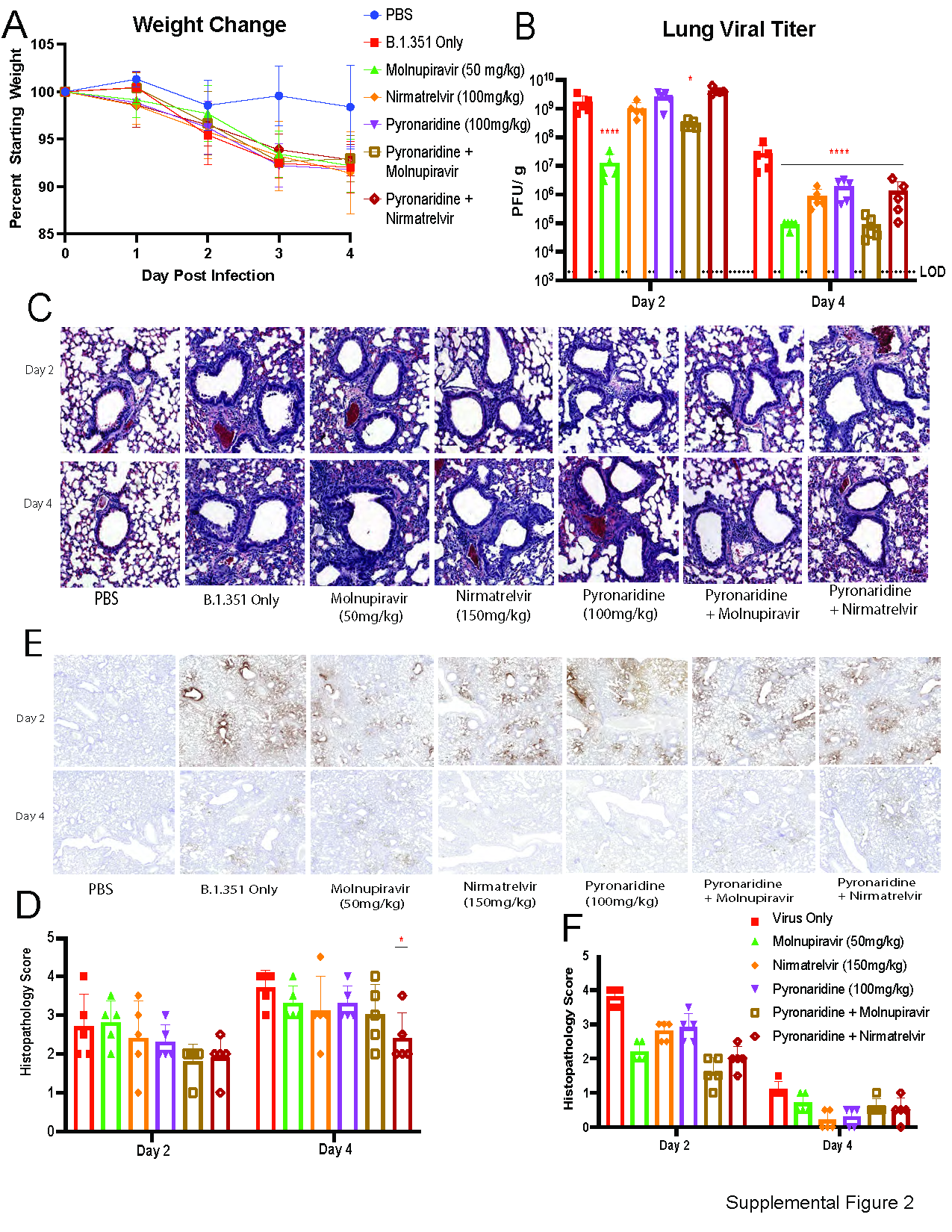

Supplement: Fig. S2 — Related to Fig. 2 and 3. [file mbio.01587-23-s0003.tif]

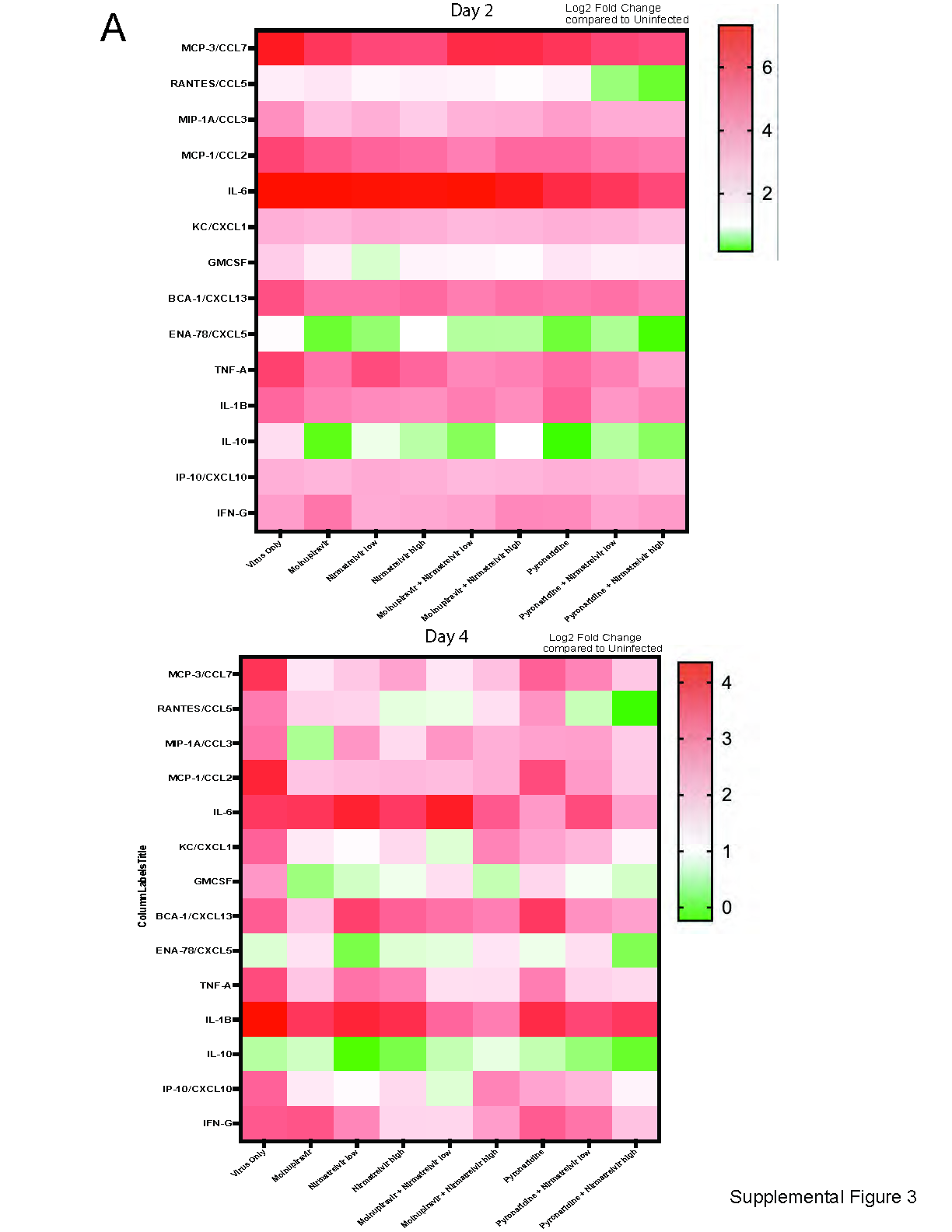

Supplement: Fig. S3 — Related to Fig. 2 and 3. [file mbio.01587-23-s0005.tif]

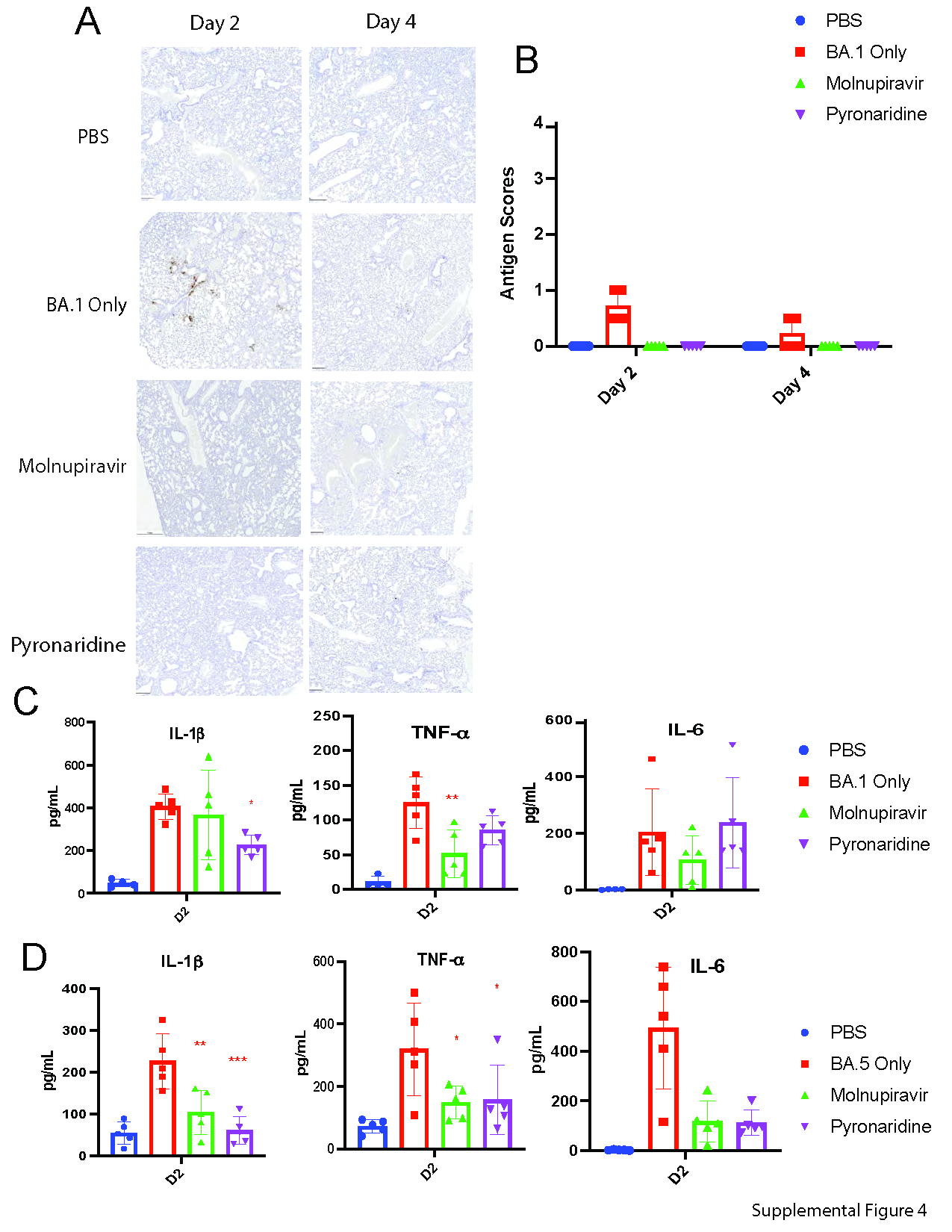

Supplement: Fig. S4 — Related to Fig. 5. [file mbio.01587-23-s0007.tif]
